# Hygiene behaviours and protective attitudes in haemodialysis patients during COVID-19: impact on quality of life

**DOI:** 10.1186/s12882-026-04768-6

**Published:** 2026-02-02

**Authors:** Sukran Bicakci, Silva Polat Sari, Mehtap Sonmez, Reyhan Caliskan

**Affiliations:** 1 Vocational School of Health Services, Dialysis Programme, Istanbul Aydin University, Istanbul, 34295 Türkiye; 2 Vocational School of Health Services, Medical Laboratory Techniques Programme, Istanbul Aydin University, Istanbul, 34295 Türkiye; 3 Department of Nursing, Faculty of Health Sciences, Kahramanmaras Sutcu Imam University, Kahramanmaras, 46100 Türkiye; 4 Department of Medical Microbiology, Faculty of Medicine, Samsun University, Samsun, 55080 Türkiye

**Keywords:** COVID-19, Health behavior, Infection control, Psychosocial factors, Quality of life, Renal dialysis

## Abstract

**Background:**

The COVID-19 pandemic underscored the critical importance of hygiene, particularly among high-risk populations such as haemodialysis patients. Maintaining strict hygiene and infection-control measures is therefore vital for this group. However, excessive hygiene routines may also affect patients’ daily lives and overall well-being. In this study aims to determine the personal protective attitudes of hemodialysis patients during the COVID-19 pandemic and the impact of increased hygiene behaviors on quality of life.

**Methods:**

A cross-sectional study was conducted between December 2021 and January 2022 in three dialysis centres across Türkiye with 383 adult haemodialysis patients. Data were collected face-to-face interviews using the COVID-19 Hygiene Scale and the COVID-19 Impact on Quality of Life Scale. Statistical analyses included Chi-square, Kruskal–Wallis, Mann–Whitney U, and Pearson correlation tests, with *p* < 0.05 considered significant.

**Results:**

Female patients scored significantly higher than males in “changed hygiene behaviours”, “home hygiene”, and “hygiene after returning home”. Participants younger than 65 years showed greater adherence to hygiene practices after returning home. Higher income levels were associated with better compliance with “social distancing and mask use” and “shopping hygiene”. There was a negative correlation between the quality of life scale and hygiene behaviors.

**Conclusions:**

Haemodialysis patients demonstrated strong adherence to hygiene measures during the COVID-19 pandemic; however, excessive hygiene behaviours were negatively associated with quality of life. These findings highlight the importance of adopting balanced, patient-centred infection-control strategies that support both behavioural adherence and overall well-being.

**Clinical trial number:**

Not applicable.

**Supplementary Information:**

The online version contains supplementary material available at 10.1186/s12882-026-04768-6.

## Introduction

The novel coronavirus, which first emerged in China in December 2019, spread rapidly around the world, posing a global threat to public health [1, 2]. The severity of the disease, its lethality, and its impact on vulnerable groups have frightened the entire world, bringing protective measures to the forefront [3, 4]. To mitigate the spread of the virus, comprehensive public health measures were implemented globally, including the use of face masks, social distancing, hand hygiene, isolation protocols, and lockdown restrictions [5, 6]. These interventions not only contributed to the containment of COVID-19 but also significantly reduced the incidence of other infectious diseases, including influenza, respiratory syncytial virus (RSV), and norovirus. However, the costs that countries had to allocate for diagnosis, treatment, and prevention during the COVID-19 pandemic have been challenging for them [7–10].

The pandemic period led to heightened awareness of hygiene and preventive health behaviours across society. Notable behavioural changes were observed, particularly regarding face mask usage, handwashing practices, and the isolation of symptomatic individuals [5, 6]. Compared to the pre-pandemic period, preventive measures such as mask-wearing, enhanced hand hygiene, and staying at home were reported to have become more widespread during the pandemic [11]. These behavioural adaptations are of critical importance, especially for individuals with compromised immune systems or underlying chronic conditions, such as patients undergoing maintenance haemodialysis. Studies have reported that compliance with pandemic rules varies depending on the sociodemographic characteristics and anamnesis of the society. A study conducted in Iran revealed that there are misconceptions about the coronavirus. This is attributed to factors such as limited access to scientific information and healthcare, inconsistent information circulating among the public from unreliable sources, lack of in-person education, limited economic resources, and cultur [12]. Burnout among healthcare workers and nurses in establishing a safety culture is also a factor that will affect this situation [13, 14]. The impact of COVID-19 on the world and societies is more critical than anticipated and these crises require greater public attention and urgent mobilization. Taking protective measures is as important in preventing the spread of the virus as it is in reducing unnecessary activities [15]. Unlike other studies in the literature (e.g., studies on peritoneal dialysis or the general population) this study is help us understand the extent to which hemodialysis patients comply with these rules [5–17].

Chronic kidney disease (CKD) is a serious lifelong condition associated with high morbidity and mortality and requires continuous medical treatment. The majority of individuals with CKD depend on regular haemodialysis sessions to maintain life. Due to their need to attend healthcare facilities several times a week, haemodialysis patients represent a high-risk group for exposure to infectious diseases during the pandemic. Furthermore, haemodialysis centres are typically enclosed and shared spaces, which increases the risk of transmission of airborne pathogens such as SARS-CoV-2. The advanced age and high burden of comorbidities commonly observed in this patient population render them particularly vulnerable to severe COVID-19 outcomes [16–18].

Beyond general public health measures, hemodialysis centers had to rapidly adapt their operational routines to protect patients with end-stage renal disease (ESRD) from infection. In response to these risks, numerous infection-control strategies were implemented worldwide to reduce transmission among hemodialysis patients. These included reorganizing dialysis schedules to create additional or staggered shifts, limiting the number of patients dialyzing simultaneously, maintaining at least 6 feet of distance between dialysis stations, and shortening session durations when feasible [19, 20]. Universal masking, temperature screening, and strict hand hygiene were mandated, and staff were required to use personal protective equipment (PPE) at all times [21, 22]. In Türkiye, the Turkish Society of Nephrology issued national guidance emphasizing pre-session triage, isolation or cohorting of suspected or confirmed cases, and the reorganization of patient transport and waiting areas to reduce contact [23]. These preventive measures aimed to mitigate the intrinsic risk of in-center hemodialysis units, where maintaining social distancing is inherently challenging. Another study in the literature that people’s failure to adhere to the health protocols established by the national centre to combat this disease is primarily attributed to administrative, individual, social, media, economic, and cultural factors, and this order is considered to be valid [24]. Risk perception during a pandemic may be critical for compliance with protective measures and administrative planning [25].

Despite these measures, hemodialysis patients continued to experience substantial challenges during the pandemic. In a case report about dialysis patients who contracted COVID-19, it was reported that in addition to being at greater risk due to their fragility, the symptoms seen in these patients were not distinctive and were confused with symptoms commonly seen in dialysis patients, which was a significant challenge [21]. The effects of COVID-19 on hemodialysis patients have been reported in the literature through small case reports. Furthermore, mortality among COVID-19 patients requiring maintenance hemodialysis has been reported to be high (6.5–52%) [22, 23, 26]. In another study, the mortality rate in hemodialysis patients compared to the general population was reported as 30.5% [27]. Another study showed that the mortality of hemodialysis populations infected with COVID-19 is 28.70% [28]. Because of the difficulty in diagnosing COVID-19 in hemodialysis patients and the increase in respiratory problems and the further criticality of their general condition, taking protective measures and differential diagnosis are of vital importance.

Health-related behaviours, including adherence to hygiene and preventive measures, are shaped by individuals’ perceptions of illness risk and the benefits or barriers of protective actions, as outlined in the Health Belief Model (HBM) [29]. This framework helps explain why certain demographic groups may demonstrate stronger engagement in preventive behaviours—particularly among vulnerable populations like haemodialysis patients during the COVID-19 pandemic.

Recent studies have shown that the long-term symptom burden among hemodialysis patients with COVID-19 was higher than that of the general population, highlighting the need to develop effective management strategies tailored to this vulnerable group [30, 31]. The biosocial environment of the pandemic has increased the vulnerability of individuals with chronic illnesses, such as dialysis patients. The pandemic has empowered and changed individuals to make choices and take action by influencing their adaptive capacities and access to available measures [32].

In this context, the present study aims to determine the personal protective attitudes of hemodialysis patients during the COVID-19 pandemic and the impact of increased hygiene behaviors on their quality of life.

## Materials and methods

### Study design

This study was designed as a cross-sectional analytic survey to evaluate hygiene behaviours and individual protective measures among patients receiving haemodialysis treatment during the COVID-19 pandemic. The most appropriate research method is one that is cross-sectional, as this allows us to explain the knowledge, attitudes and practices (KAP) of patients. Ethical approval for this study was obtained from the Non-Interventional Clinical Research Ethics Committee of Istanbul Aydın University (Decision No: 2021/638), following permission granted by the Scientific Research Platform of the Ministry of Health for conducting research on COVID-19.

### Participants

The first cases of the pandemic in Türkiye were announced on March 11, 2020, and 2021 is a period when data on increased deaths were announced and protective measures and immunization were implemented. The study was conducted between December 2021 and January 2022 at three dialysis centres in Istanbul. There was total 128 Dialysis Center in Istanbul. This centers was selected sample random method. The research was carried out under the coordination of Istanbul Aydın University. Inclusion criteria comprised individuals aged 18 years or older, currently receiving haemodialysis treatment, literate in Turkish, and voluntarily consenting to participate in the study. Based on using the unknown sampling method of the universe conducted with a 95% confidence interval and a 5% margin of error, the minimum required sample size was calculated to be 383 participants. All participants was selected sample random method according to the patient list in this centers.

### Data collection

Data were collected through face-to-face interviews using a structured questionnaire comprising three main sections:

1. **Personal Information Form**: This section collected data on participants’ demographic characteristics and health status.
2. **COVID-19 Hygiene Scale**: A validated and reliable scale developed by Çiçek et al. in 2020 to assess hygiene behaviours during the pandemic [33]. It is a 27-item scale consisting of 6 sub-dimensions. “Changing Hygiene Behaviors” (Items 7, 11, 12, 14, 21, and 27), “Home Hygiene” (Items 16, 18, 19, and 20), “Social Distancing and Mask Use” (Items 1, 2, 3, and 25), “Shopping Hygiene” (Items 15, 22, 23, 24, and 26), “Hand Hygiene” (Items 4, 5, 6, 8, and 9), and “Hygiene When Coming Home from Outside” (Items 10, 13, and 17). This scale cronbach alpha 0.96.
3. **Impact of COVID-19 on Quality of Life Scale**: A validated and reliable instrument developed by Sümen and Adibelli in 2021 to evaluate the perceived effect of the pandemic on quality of life [34]. The scale score is calculated by dividing the total score by the number of items. A higher score indicates a greater impact of the pandemic on a person’s quality of life. This scale cronbach alpha was 0,91.

The aim and procedures of the study were thoroughly explained to all participants, and written informed consent was obtained.

### Statistical analysis

The required sample size was determined by statistical power analysis, and the final sample consisted of 383 patients. Complete and accurate data were included in the research. There was no missing data. Data obtained from the study were analysed using IBM SPSS Statistics version 25.0. The normality of the data distribution was assessed using the Kolmogorov-Smirnov test The measurable data were not normally distributed and the variances were not homogeneous. We also checked the skewness and kurtosis values ​​and the data did not show a normal distribution. For categorical variables, the Chi-square test was employed, while continuous variables were analysed using non-parametric tests. The Kruskal-Wallis and Mann-Whitney U tests were used for non-normally distributed data. Post Hoc test (Tukey and dunn tests) was performed to find the groups that constitute significance. A p-value of less than 0.05 was considered statistically significant, with a confidence level of 95%.

## Ethical consideration

Ethical approval was obtained from the ethics committee of Istanbul Aydin University. Participants were included in the study after providing written and verbal consent. Data were collected in this study in accordance with the Helsinki Declaration. All procedures performed in this study involving human participants were conducted in accordance with the ethical standards of the institutional and/or national research committee. The de-identified dataset is available from the corresponding author upon reasonable request.

## Results

Some variables of hemodialysis patients included in this study are presented in Table 1.

**Table 1:** Some variables of hemodyalisis patients included in this study

Variables	n	%	Variables	n	%
Groups of Age			COVID 19 Status		
< 65	208	54.3	Yes	99	25.8
65 ve üzeri	175	45.7	No	284	74.2
Gender			Knowing the Way of COVID 19 Transmission		
Female	165	43.1	Yes	376	98.2
Male	218	56.9	No	7	1.8
Income Status*			COVID 19 Vaccine Status		
Low	44	11.5	Yes	360	94.0
Middle	312	81.5	No	23	6.0
High	27	7.0	Sinovac Vaccine Status		
Status of Education			Yes	284	74.2
Primary	290	75.7	No	99	25.8
High School	53	13.8	Biontech Vaccine Status		
Comorbidities Status			Yes	157	41.0
Yes	247	64.5	No	226	59.0
No	136	35.5	Ways of protection	n	%
Hepatitis B			Mask Use	19	5.0
Yes	10	2.61	Mask Use + Hand Washing	29	7.6
No	373	97.3	Mask Use + Hand Washing + Disinfection	217	56.7
Feeling anxious about one's health			Mask Use + Hand Washing + Disinfection + Glove use	118	30.8
Yes	164	42.8			
No	124	32.4			
Indecisive	95	24.8			
Total	383	100.0	Total	383	100.0

The study was completed with a total of 383 participants. Of the participants, 56.9% were male and 43.1% were female. The mean age was calculated as 62.18 ± 15.03 years, with a range from 19 to 92 years. Among the individuals included in the study, 64.5% had at least one comorbid condition in addition to kidney failure, with hypertension being the most common (40.9%). The rate of those who are feeling anxious about their health is 42.8%.

Only statistically significant results are presented in the main text. The mean scale scores of women, those over 65 years of age, those who are Hepatitis B negative, those with chronic diseases, those who have received the Sinovac vaccine and those who have not received the BionTech vaccine were found to be higher than the other groups when the scale was compared with some sociodemographic characteristics, although not significantly.

A full summary of all subscale scores and comparative analyses, including non-significant findings, is provided in Supplementary File. A comparison of some data with various sub-dimensions of COVID-19 hygiene behaviors is shown in Table 2.

**Table 2:** Comparison of sociodemographic variables by subdimensions of the COVID-19 hygiene scale

Subdimensions	Gender (Female/Male)	Age (< 65 / ≥65)	Income Level (Low / Medium / High)
Changed Hygiene Behaviours	20.77 / 19.46 (*p* = 0.009)	20.59 / 19.75 (*p* = 0.310)	19.86 / 19.93 / 21.93 (*p* = 0.349)
Home Hygiene	**14.45 / 13.20 **(p = 0.001)*	13.88 / 13.57 (*p* = 0.403)	14.15 / 13.61 / 14.11 (*p* = 0.593)
Social Distancing and Mask Use	15.54 / 15.22 (*p* = 0.350)	15.36 / 15.36 (*p* = 0.987)	13.90 / 12.62 / 14.48 (p = 0.004)*
Shopping Hygiene	14.06 / 13.75 (*p* = 0.610)	14.32 / 13.37 (*p* = 0.113)	11.68 / 14.04 / **15.70** (p = 0.015)*
Hygiene After Returning Home	**10.66** / 9.95 **(** p = 0.012)*	**10.56** / 9.89 **(**p = 0.015)*	10.68 / 10.22 / 9.98 (*p* = 0.551)
Hand Hygiene	19.09 / 18.56 (*p* = 0.271)	18.87 / 18.69 (*p* = 0.706)	18.79 / 18.28 / 20.25 (*p* = 0.191)

*p < 0,05, ıt analyzed by post hoc test (Tukey and dunn tests: Gender; female, < 65 age, High income). The groups constituting significance are shown in bold

Female participants scored significantly higher than males in the subdimensions of “changed hygiene behaviours”, “home hygiene”, and “hygiene after returning home” (*p* = 0.009, *p* = 0.001, and *p* = 0.012, respectively). Although both genders demonstrated high levels of “hand hygiene” behaviour, no statistically significant difference was found between them (Table 2). In the subdimension of “hygiene after returning home”, individuals under the age of 65 scored significantly higher than those aged 65 and over (*p* = 0.012). However, no statistically significant differences were observed between age groups in the other subdimensions (Table 2). Statistically significant differences were found in the subdimensions of “social distancing and mask use” and “shopping hygiene” according to income level. It was observed that these preventive behaviours were more frequently practiced as income level increased (*p* = 0.004, *p* = 0.015) (Table 2).

According to the results of the Impact of COVID-19 on Quality of Life Scale, an increase in hygiene measures was associated with a decrease in quality of life. Notably, negative correlations were identified between quality of life and the subdimensions of “changed hygiene behaviours” (*r* = − 0.185; *p* = 0,000), “home hygiene” (*r* = − 0.169; *p* = 0.000), and “hygiene after returning home” (*r* = − 0.100; *p* = 0,050) (Table 3).

**Table 3:** Correlations between subdimensions of the COVID-19 hygiene scale and quality of life

Subdimensions	Correlation Coefficient (*r*)	*p*-value
Changed Hygiene Behaviors	-0.185**	0.000
Home Hygiene	-0.169**	0.000
Social Distancing and Mask Use	0.004	0.941
Shopping Hygiene	-0.066	0.200
Hygiene After Returning Home	-0.100*	0.050
Hand Hygiene	-0.055	0.282

Pearson Correlation analysis **p < 0.01 level high significantly, *p < 0.05 level middle significantly

## Discussion

The present study aimed to investigate hygiene behaviours among haemodialysis patients during the pandemic period and examine the impact of these behaviours on their quality of life. It found that more than half of the heamodialysis patients were male, and their average age was close to 65. Over half of the patients had more than one chronic disease. This placed them in the high-risk group for the pandemic.

The COVID-19 pandemic has led to significant global changes in individuals’ hygiene behaviours. Individuals with compromised immune systems or chronic illnesses, olderly person, comorbidite were identified as high-risk groups due to their increased vulnerability to severe outcomes of the infection. Patients undergoing haemodialysis are particularly susceptible to infections owing to their weakened immune systems, the presence of comorbidities, and the necessity of frequent visits to healthcare facilities [18]. This creates patient profile that requires stricter adherence to hygiene protocols. The death rate of 25.6% to 30% seen in patients from haemodialysis centres not using pandemic protocols. In addition, structural and organizational changes, as well as patient compliance, have played a significant role in controlling the spread of the disease and increasing survival rates [35].

When analysed in terms of gender differences, our findings are consistent with those of Aydın et al., who conducted a community-based study in Türkiye during the early phase of the pandemic, and reported that female participants had significantly higher total scores, as well as higher scores in subdimensions such as hand hygiene, social distancing and mask use, and home hygiene compared to males and in another study by Çicek et al., general hygiene behaviorus of men were inadaquete [33, 36]. Both studies were performed in the general population, yet our results indicate that this gender pattern persists even in high-risk groups such as haemodialysis patients, who are subject to stricter infection control routines. Although the present study did not directly employ a theoretical model, these gender-based differences can be interpreted within the framework of the Health Belief Model. According to this model, women’s higher perceived susceptibility to illness and stronger belief in the benefits of preventive behaviours may partly explain their greater adherence to hygiene practices [29]. In another study was that reported to men, women demonstrated a heightened awareness of the pandemic’s potential risks. It shown to men, women were also more likely to adopt health-protective behaviours such as wearing face masks, washing their hands, avoiding public places and practising sanitisation [37].

In terms of age, the study by Aydın et al. reported that individuals in younger age groups placed greater emphasis on hand hygiene [36]. Similarly, a study conducted in Poland by Glabska et al. among students aged 15–20 found that the frequency of handwashing increased during the COVID-19 pandemic, with significant behavioural changes observed particularly in hygiene practices after returning home [38]. Unlike these studies, which focused on adolescents and healthy adults, our study included middle-aged and elderly haemodialysis patients. Nevertheless, the finding that individuals under 65 scored higher in the “hygiene after returning home” subdimension suggests that more younger adults, even within this clinical population, remain more behaviourally adaptive and socially aware of infection risks. This result may be interpreted as a reflection of the heightened perception of infection risk among younger and more socially active individuals, who are more frequently involved in social life and therefore tend to adopt hygiene measures more consistently. In contrast, the lower scores observed among patients aged 65 and older may be associated with age-related physical and cognitive limitations, such as reduced mobility, visual or hearing impairment, and difficulties in maintaining consistent hygiene routines. This multifactorial background may explain the relatively lower hygiene scores observed among elderly haemodialysis patients. In addition, the low level of education (75.7%) of most patients may also be a factor affecting this situation.

On the other hand, in a study conducted by Uğurlu-Kalkan et al. involving individuals aged 18 to 70, only 57.7% of participants reported washing their hands 11 times or more per day [39]. This study was conducted in the general population rather than in patients with chronic diseases, which may partly explain the lower levels of hand hygiene reported. In contrast, haemodialysis patients, who are regularly exposed to healthcare environments and infection control protocols, may have developed a stronger routine adherence to hygiene practices. Many studies in different populations have reported that excessive handwashing causes contact dermatitis, and that patients with high anxiety levels wash their hands frequently [40–42]. Therefore, in addition to age, individual awareness, disease-related experience, and health literacy also emerge as significant determinants of hygiene behaviours. During risky periods, such as during the current global pandemic of the COVID-19, anxiety-managed protective models can be effective in preventing excessive behaviours that may develop in haemodialysis patients. Studies in the literature indicate that establishing institutional protocols during the COVID-19 pandemic fosters a safety culture for both healthcare professionals and patients. Furthermore, the effectiveness of patient education in improving compliance with measures is emphasized, and it is recommended that the frequency of patient education be increased. It has also been reported that the use of health technologies such as telemedicine applications and mobile education has been effective in the COVID-19 pandemic [43–45].

Current study reported significant differences related income levels. In addition scores for changing hygiene behaviours, shopping hygiene, and hand hygiene were found that to tbe greater high income (in Table 2) in other studies revealed that scores for “social distancing and mask use” and “shopping hygiene” increased with higher income levels [36]. Higher income levels may facilitate better access to hygiene supplies, private transportation, and safer living conditions, all of which contribute to stricter adherence to preventive measures. This finding suggests that socioeconomic status may directly influence engagement in health-protective behaviours, particularly among individuals with chronic illnesses who depend on regular healthcare services.

When evaluating the impact of hygiene behaviours on quality of life, our results indicated that individuals who scored higher in the subdimensions of “changed hygiene behaviours”, “home hygiene”, and “hygiene after returning home” reported lower quality of life scores. This suggests that strict adherence to hygiene measures during the pandemic may have imposed psychological and physical burdens, thereby negatively affecting overall quality of life. However, our participants were haemodialysis patients who were already coping with chronic illness and treatment-related stress, which may have amplified the psychosocial strain associated with strict hygiene routines. Participants in our study likely experienced heightened anxiety due to the increased hygiene burden and social isolation during the pandemic, leading to weakened social relationships, reduced sense of autonomy, and lower satisfaction with their environment.

Conversely, a study conducted by Bang et al. in South Korea reported that, despite challenges in maintaining personal hygiene and social distancing, such behaviours were associated with a sense of protection and responsibility, thereby positively contributing to quality of life [46]. Likewise, in a study by Czabanowska et al. involving medical students in Poland, a positive association was found between protective hygiene behaviours and quality of life, with “positive mental attitude” identified as one of the strongest predictors of well-being [47]. These divergent findings indicate that the impact of hygiene behaviours on quality of life may vary depending on individual factors such as health status, age, educational level, and personal interpretation of the pandemic. While hygiene practices are undoubtedly important for public health, their sustainability, balance, and compatibility with the individual’s psychosocial context appear to be critical determinants of quality of life. The risk of COVID-19 spreading to other patients in the community, reduced social interaction, and psychosocial problems such as isolation, panic, fear of death, and stigma affect the process of compliance with protective measures [48].

In general, studies in the literature have observed an increase in hygiene behaviors and in reducing the incidence of other infectious diseases during COVID-19 lockdown [7–9, 49]. While most of these studies were conducted in peritoneal dialysis populations, their findings collectively highlight how the pandemic catalyzed behavioural change and infection-control awareness among dialysis patients, a trend also evident in our haemodialysis cohort. Sustaining these practices may not only protect individual health but also help alleviate the burden on healthcare systems.

Many factors, such as taking multiple medications, maintaining a healthy diet, and adhering to hygiene standards, can hinder compliance with daily life activities for hemodialysis patients. In addition to this normal situation, maintaining protective measures during the COVID-19 pandemic impacts compliance with excessive hygiene practices. These effects, albeit indirectly, can lead to changes in the patient’s general condition, prolonged symptom burden, and increased psychological and economic burden. Hemodialysis is a treatment process that reduces patients’ quality of life and is associated with a significant symptom burden. It has been observed that hemodialysis patients who have had COVID-19 experience a greater symptom burden, and patients undergoing hemodialysis experience longer-term symptoms during the COVID-19 period [50]. It has been stated that a patient-centered nursing approach that considers individual differences and new strategies in care management, rather than traditional and uniform interventions, are crucial for improving quality of life and reducing disease burden.

The study has certain limitations. The data for this study were collected face-to-face during dialysis sessions with hemodialysis patients. It was conducted in only three dialysis centres, which may limit the generalisability of the findings. Additionally, participants’ mental health status was not assessed, which could have provided deeper insights into the psychosocial dimensions of quality of life. Cross sectional design limits causal inference and future longitudinal, multicentre studies with larger sample sizes are needed to validate these findings and inform the development of more comprehensive intervention models for infection control and patient well-being. This study was conducted on hemodialysis patients diagnosed with COVID-19, following Turkish Health Services protocols. The study is limited by the fact that each country’s COVID-19 protocols are specific to its specific country.

## Conclusion

As a result, female gender, younger age, and higher income levels were found to be associated with more frequent and consistent engagement in hygiene-related behaviours. However, increased scores in subdimensions such as “changed hygiene behaviours,” “home hygiene,” and “hygiene after returning home” were negatively correlated with quality of life. Considering the impact of short-term anxiety screening on health education and adherence to preventive measures for vulnerable groups, it is recommended to increase patient-centered training and establish peer support groups. These findings suggest that infection control strategies should not solely focus on protecting physical health but must also incorporate holistic approaches that consider individuals’ psychological and social well-being. Therefore, preventive health services should aim not only to promote behavioural change but also to develop supportive policies that minimise the potential psychological toll of these behaviours on individuals. Stress and anxiety coping interventions model, HBM, integrating psychological support and behavioural education into dialysis care protocols could help patients sustain effective hygiene habits while preserving mental well-being and overall quality of life. In the future, it is recommended that new studies be conducted to monitor the health behaviors of chronic patients in situations of uncertainty and to develop models for critical situations.

## Supplementary Information

Below is the link to the electronic supplementary material.

Supplementary Material 1

## Data Availability

All data generated or analyzed during this study are included in the supplementary information file attached to this article.

## Declarations

### Artifical intelligience politics

There was no AI in this study.
