# Supplementary material for: Hygiene behaviours and protective attitudes in haemodialysis patients during COVID-19: impact on quality of life
Source: BMC Nephrol. 2026 Feb 2;27:148. doi: 10.1186/s12882-026-04768-6 (PMC12952057; doi:10.1186/s12882-026-04768-6)
Supplement: Supplementary file 1 — Supplementary Material 1 [file 12882_2026_4768_MOESM1_ESM.docx]

Table 1. Relationship Between Gender and Subdimensions of the COVID-19 Hygiene Scale

| **COVID-19 Hygiene Subdimensions** | **Female (Mean ± SD)** | **Male (Mean ± SD)** | **Z / p-value** |
| --- | --- | --- | --- |
| Changed Hygiene Behaviours | 20.77 ± 4.83 | 19.46 ± 4.80 | 2.642 / **0.009*** |
| Home Hygiene | 14.45 ± 3.59 | 13.20 ± 3.59 | 3.371 / **0.001*** |
| Social Distance – Mask Use | 15.54 ± 3.21 | 15.22 ± 3.40 | 0.935 / 0.350 |
| Shopping Hygiene | 14.06 ± 5.82 | 13.75 ± 5.93 | 0.510 / 0.610 |
| Hygiene After Returning Home | 10.66 ± 2.61 | 9.95 ± 2.75 | 2.539 / **0.012*** |
| Hand Hygiene | 19.09 ± 4.69 | 18.56 ± 4.51 | 1.102 / 0.271 |

****p < 0.05, Mann–Whitney U test.***

Table 2. Relationship Between Age and Subdimensions of the COVID-19 Hygiene Scale

| **COVID-19 Hygiene Subdimensions** | **<65 Years (Mean ± SD)** | **≥65 Years (Mean ± SD)** | **Z / p-value** |
| --- | --- | --- | --- |
| Changed Hygiene Behaviours | 20.59 ± 5.08 | 19.75 ± 4.55 | 1.016 / 0.310 |
| Home Hygiene | 13.88 ± 3.77 | 13.57 ± 3.48 | 0.830 / 0.403 |
| Social Distance – Mask Use | 15.36 ± 3.47 | 15.36 ± 3.14 | 0.016 / 0.987 |
| Shopping Hygiene | 14.32 ± 5.95 | 13.37 ± 5.76 | 1.586 / 0.113 |
| Hygiene After Returning Home | 10.56 ± 2.72 | 9.89 ± 2.67 | 2.439 / **0.015*** |
| Hand Hygiene | 18.87 ± 4.72 | 18.69 ± 4.44 | 0.377 / 0.706 |

****p < 0.05, Mann–Whitney U test.***

Table 3. Relationship Between Income Level and Subdimensions of the COVID-19 Hygiene Scale

| **COVID-19 Hygiene Subdimensions** | **Low**  **(Mean ± SD)** | **Medium**  **(Mean ± SD)** | **High**  **(Mean ± SD)** | **χ² / p-value** |
| --- | --- | --- | --- | --- |
| Changed Hygiene Behaviours | 19.86 ± 3.65 | 19.93 ± 5.12 | 21.93 ± 3.61 | 1.056 / 0.349 |
| Home Hygiene | 14.15 ± 2.62 | 13.61 ± 3.84 | 14.11 ± 2.45 | 0.524 / 0.593 |
| Social Distance – Mask Use | 13.90 ± 3.33 | 14.48 ± 2.30 | 12.62 ± 3.32 | 5.624 / **0.004*** |
| Shopping Hygiene | 11.68 ± 4.67 | 14.04 ± 5.90 | 15.70 ± 5.55 | 4.571 / **0.015*** |
| Hygiene After Returning Home | 10.68 ± 2.15 | 10.22 ± 2.82 | 9.98 ± 2.25 | 0.755 / 0.551 |
| Hand Hygiene | 18.79 ± 4.72 | 18.28 ± 2.25 | 20.25 ± 4.58 | 1.661 / 0.191 |

****p < 0.05, Kruskal–Wallis test.***

Table 4. Demographic Characteristics and COVID-19 Quality of Life Scale Scores

| **Variable** | **Group** | **Mean ± SD** | **Test** | **p-value** |
| --- | --- | --- | --- | --- |
| Gender | Female | 2.95 ± 0.91 | 0.206* | 0.231 |
|  | Male | 2.81 ± 0.91 |  |  |
| Age | <65 years | 2.86 ± 0.94 | 0.241* | 0.624 |
|  | ≥65 years | 2.91 ± 0.89 |  |  |
| History of COVID-19 | Yes | 2.88 ± 0.96 | 1.538* | 0.970 |
|  | No | 2.88 ± 0.90 |  |  |
| Hepatitis B | Yes | 2.89 ± 0.92 | 1.722* | 0.930 |
|  | No | 2.73 ± 0.78 |  |  |
| Chronic Disease | Yes | 2.88 ± 1.04 | 0.672* | 0.557 |
|  | No | 2.69 ± 0.91 |  |  |
| Sinovac Vaccination | Yes | 2.90 ± 0.90 | 0.409* | 0.246 |
|  | No | 2.88 ± 0.92 |  |  |
| Biontech Vaccination | Yes | 2.80 ± 0.91 | 0.000* | 0.989 |
|  | No | 2.88 ± 0.91 |  |  |

****p < 0.05. Kruskal-Wallis Test; Mann–Whitney U Test.***

Table 5. Relationship Between Gender and Quality of Life Scores Related to COVID-19

| **Scale Score** | **Gender** | **n (%)** | **Chi-Square / p-value** |
| --- | --- | --- | --- |
| <2 points | Female | 34 (39.5) | 0.893 / 0.827 |
|  | Male | 52 (60.5) |  |
| 2.1–3.0 | Female | 59 (43.4) |  |
|  | Male | 77 (56.6) |  |
| 3.1–4.0 | Female | 55 (43.7) |  |
|  | Male | 71 (56.3) |  |
| 4.1–5.0 | Female | 17 (48.6) |  |
|  | Male | 18 (51.4) |  |
| Total | Female | 165 (43.1) |  |
|  | Male | 218 (56.9) |  |

***Chi-square test, p < 0.05 considered statistically significant.***

Table 6. Effect of COVID-19 Infection History on Quality of Life Scores

| **Scale Score** | **COVID-19 Infectionn**  **Yes**  **n(%)** | **COVID-19 Infectionn**  **No**  **n(%)** | **Chi-Square / p-value** |
| --- | --- | --- | --- |
| <2 points | 24 (27.9) | 62 (72.1) | 1.154 / 0.764 |
| 2.1–3.0 | 32 (23.5) | 104 (76.5) |  |
| 3.1–4.0 | 32 (25.4) | 94 (76.4) |  |
| 4.1–5.0 | 11 (31.4) | 24 (68.6) |  |
| Total | 99 (25.8) | 284 (74.2) |  |

***Chi-square test, p < 0.05 considered statistically significant.***

Table 7. Association Between COVID-19 Vaccination Status and Quality of Life Scores

| **Scale Score** | **COVID-19 Vaccination Status** | **COVID-19 Vaccination Status** | **Chi-Square / p-value** |
| --- | --- | --- | --- |
|  | **Yes**  **n(%)** | **No**  **n(%)** |  |
| <2 points | 80 (93.0) | 6 (7.0) | 0.198 / 0.978 |
| 2.1–3.0 | 128 (94.1) | 8 (5.9) |  |
| 3.1–4.0 | 119 (94.4) | 7 (5.6) |  |
| 4.1–5.0 | 33 (94.3) | 2 (5.7) |  |
| Total | 360 (94.0) | 23 (6.0) |  |

***Chi-square test, p < 0.05 considered statistically significant.***

Table 8. Association Between COVID-19 Vaccination Status and History of Infection

| **COVID-19 Vaccination Status** | **History of COVID-19 Infection** | **n (%)** | **Chi-Square / p-value** |
| --- | --- | --- | --- |
| Yes | Yes | 93 (25.8) | 1.000 / 0.573 |
|  | No | 267 (74.2) |  |
| No | Yes | 6 (26.1) |  |
|  | No | 17 (73.9) |  |
| Total | Yes | 99 (25.8) |  |
|  | No | 284 (74.2) |  |

***Chi-square test, p < 0.05 considered statistically significant.***
